# Hierarchical Anatomical Brain Networks for MCI Prediction: Revisiting Volumetric Measures

**DOI:** 10.1371/journal.pone.0021935

**Published:** 2011-07-19

**Authors:** Luping Zhou, Yaping Wang, Yang Li, Pew-Thian Yap, Dinggang Shen, and the Alzheimer's Disease Neuroimaging Initiative (ADNI)

**Affiliations:** 1 IDEA Lab, Department of Radiology and BRIC, University of North Carolina, Chapel Hill, North Carolina, United States of America; 2 Alzheimer's Disease Neuroimaging Initiative, Department of Neurology, University of California Los Angeles, Los Angeles, California, United States of America; University of Muenster, Germany

## Abstract

Owning to its clinical accessibility, T1-weighted MRI (Magnetic Resonance Imaging) has been extensively studied in the past decades for prediction of Alzheimer's disease (AD) and mild cognitive impairment (MCI). The volumes of gray matter (GM), white matter (WM) and cerebrospinal fluid (CSF) are the most commonly used measurements, resulting in many successful applications. It has been widely observed that disease-induced structural changes may not occur at isolated spots, but in several inter-related regions. Therefore, for better characterization of brain pathology, we propose in this paper a means to extract inter-regional correlation based features from local volumetric measurements. Specifically, our approach involves constructing an anatomical brain network for each subject, with each node representing a Region of Interest (ROI) and each edge representing Pearson correlation of tissue volumetric measurements between ROI pairs. As second order volumetric measurements, network features are more descriptive but also more sensitive to noise. To overcome this limitation, a hierarchy of ROIs is used to suppress noise at different scales. Pairwise interactions are considered not only for ROIs with the same scale in the same layer of the hierarchy, but also for ROIs across different scales in different layers. To address the high dimensionality problem resulting from the large number of network features, a supervised dimensionality reduction method is further employed to embed a selected subset of features into a low dimensional feature space, while at the same time preserving discriminative information. We demonstrate with experimental results the efficacy of this embedding strategy in comparison with some other commonly used approaches. In addition, although the proposed method can be easily generalized to incorporate other metrics of regional similarities, the benefits of using Pearson correlation in our application are reinforced by the experimental results. Without requiring new sources of information, our proposed approach improves the accuracy of MCI prediction from 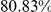 (of conventional volumetric features) to 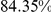 (of hierarchical network features), evaluated using data sets randomly drawn from the ADNI (Alzheimer's Disease Neuroimaging Initiative) dataset.

## Introduction

Alzheimer's disease (AD) is a progressive and eventually fatal disease of the brain, characterized by memory failure and degeneration of other cognitive functions. Pathology may begin long before the patient experiences any symptom and often lead to structural changes of brain anatomies. With the aid of medical imaging techniques, it is now possible to study in vivo the relationship between brain structural changes and the mental disorder, providing a diagnosis tool for early detection of AD. Current studies focus on MCI (mild cognitive impairment), a transitional state between normal aging and AD. These subjects suffer from memory impairment that is greater than expected for their age, but retain general cognitive functions to maintain daily living. Identifying MCI subjects is important, especially for those that will eventually convert to AD (referred to as Progressive-MCI, or in short P-MCI), because they may benefit from therapies that could slow down the disease progression.

Although T1-weighted MRI, as a diagnostic tool, is relatively well studied, it continues to receive the attention of researchers due to its easy access in clinical settings, compared with task-based functional imaging [1]. Commonly used measurements can be categorized into three groups: regional brain volumes [1]–[7], cortical thickness [8]–[12] and hippocampal volume and shape [13]–[15]. Volumetric measurements can be further divided into two groups according to feature types: voxel-based features [16] or region-based features [17], [18]. In this paper, we focus on region-based volumetric measurements of the whole brain for the following reasons. Firstly, the abnormalities caused by the disease involved in our study are not restricted to the cortex, because, as shown by pathological studies [19], AD related atrophy begins in the medial temporal lobe (MTL), which includes some subcortical structures such as the hippocampus and the amygdala. Secondly, a whole brain analysis not restricted to the hippocampus is preferred, because early-stage AD pathology is not confined to the hippocampus. Also affected are the entorhinal cortex, the amygdala, the limbic system, and the neocortical areas. As has been pointed out in several studies [1], [20], although the analysis of the earliest-affected structures, such as the hippocampus and the entorhinal cortex, can increase the sensitivity of MCI prediction, the inclusion of the later-affected temporal neocortex may increase the prediction specificity, and hence improve the overall classification accuracy [20]. Thirdly, we focus on region-based volumetric features because voxel-based features are highly redundant [21], which may affect their discrimination power.

The determination of the Region of Interest (ROI) is the key for region-based analysis methods. Once ROIs have been determined either by pre-definition [17], [18] or by adaptive parcellation [4], [5], [21], the mean tissue densities of gray matter (GM), white matter (WM) and cerebrospinal fluid (CSF) in each ROI are usually used as features for classification. Disease-induced brain structural changes may occur not at isolated spots, but in several inter-related regions. Therefore, for a more accurate characterization of the pathology, feature correlation between ROIs has to be taken into account. Measurement of such correlations may provide potential biomarkers associated with the pathology, and hence is of great research interest. However, for most existing approaches, the dependencies among features are not explicitly modelled in the feature extraction procedure, but only implicitly considered by some classifiers, such as the support vector machines (SVMs), during the classification process. For example, a linear SVM classifier models the dependency (inner product) of feature vectors between two subjects, instead of the interaction of two ROIs (via volumetric features) of a specific subject. These implicitly encoded feature dependencies become more difficult to interpret when a nonlinear SVM classifier is used. Based on this observation, we propose in this paper a new type of features derived from regional volumetric measurements, by taking into account the pairwise ROI interactions within a subject directly. To achieve this, each ROI is first characterized by a vector that consists of the volumetric ratios of GM, WM and CSF in this ROI. Then, the interaction between two ROIs within the same subject is computed as Pearson correlation of the corresponding volumetric elements. This gives us an anatomical brain network, with each node denoting an ROI and each edge characterizing the pairwise connection.

The correlation value measures the similarity of the tissue compositions between a pair of brain regions. When a patient is affected by MCI, the correlation values of a particular brain region with another region will be potentially affected, due possibly to the factors such as tissue atrophy. These correlation changes will be finally captured by classifiers and used for MCI prediction. An early work was presented in a conference [22]. It is worth noting that by computing the pairwise correlation between ROIs, our approach provides a second order measurement of the ROI volumes, in contrast to the conventional approaches that only employ first order volumetric measurement. As higher order measurements, our new features may be more descriptive, but also more sensitive to noise. For instance, the influence of a small ROI registration error may be exaggerated by the proposed network features, which may reduce the discrimination power of the features. To overcome this problem, a hierarchy of multi-resolution ROIs is used to increase the robustness of classification. Effectively, the correlations are considered at different scales of regions, thus providing different levels of noise suppression and discriminative information, which can be sieved by a feature selection mechanism as discussed below for guiding the classification. Additionally, we consider the correlations both within and between different resolution scales. This is because the optimal scale is often not known a priori. We will demonstrate the effectiveness of the proposed approach with empirical evidence. In this study, we consider a fully-connected anatomical network, features extracted from which will form a space with intractably high dimensionality. As a remedy, a supervised dimensionality reduction method is employed to embed the original network features into a new feature space with a much lower dimensionality.

Without requiring any new information in addition to the baseline T1-weighted images, the proposed approach improves the prediction accuracy of MCI from 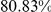 (of conventional volumetric features) to 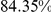 (of hierarchical network features), evaluated by data sets randomly drawn from the ADNI dataset [23]. Our study shows that this improvement comes from the use of the network features obtained from hierarchical brain networks. To investigate the generalizability of the proposed approach, experiments are conducted repetitively based on different random partitions of training and test data sets with different partition ratios. The average classification accuracy estimated in this way tends to be more conservative than the conventional Leave-One-Out approach. Additionally, although the proposed approach can be easily generalized to incorporate regional similarity measurements other than Pearson correlation, the experimental results reinforce the choice of Pearson correlation for our application, compared with some commonly used similarity metrics.

Before introducing our proposed approach, it is worth highlighting the advantages of the hierarchical brain network-based approach over the conventional volume-based approaches. Firstly, as mentioned above, our proposed method utilizes a second-order volumetric measurement that is more descriptive than the conventional first-order volumetric measurement. Secondly, compared with the conventional volumetric measurements that only consider local volume changes, our proposed hierarchical brain network considers global information by pairing ROIs that may be spatially far away. Thirdly, our proposed method seamlessly incorporates both local volume features and global network features for the classification by introducing a whole-brain ROI at the top of the hierarchy. By correlating with the whole-brain ROI, each ROI can provide a first order measurement of local volume. Fourthly, although our current approach uses Pearson correlation, it can be easily generalized to any other metrics that are capable of measuring the similarity between features of ROI pairs. Fifthly, the proposed method involves only linear methods, leading to easy interpretations of the classification results. Finally, for the first time, we investigate the *relative* speeds of disease progression in different regions, providing a different pathological perspective complementary to spatial atrophy patterns.

## Materials and Methods

### Participants

Both the normal control and MCI subjects used in the preparation of this article were obtained from the Alzheimer's Disease Neuroimaging Initiative (ADNI) database (www.loni.ucla.edu/ADNI) [23]. The ADNI was launched in 2003 by the National Institute on Aging (NIA), the National Institute of Biomedical Imaging and Bioengineering (NIBIB), the Food and Drug Administration (FDA), private pharmaceutical companies and non-profit organizations as a 60 million, 5-year public private partnership. The primary goal of ADNI has been to test whether serial MRI, PET (Positron Emission Tomography), other biological markers, and clinical and neuropsychological assessment can be combined to measure the progression of MCI and early AD. Determination of sensitive and specific markers of very early AD progression is intended to aid researchers and clinicians in the development of new treatments and monitor their effectiveness, as well as lessen the time and cost of clinical trials. The image acquisition parameters have been described in www.adniinfo.org. The ADNI protocol included a sagittal volumetric 3D MPRAGE with 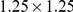 mm in-plane spatial resolution and 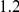-mm thick sagittal slices (8 flip angle). TR and TE values of the ADNI protocol were somewhat variable, but the target values were TE 3.9 ms and TR 8.9 ms.

The ADNI data were previously collected across 50 research sites. Study subjects gave written informed consent at the time of enrollment for imaging and genetic sample collection and completed questionnaires approved by each participating sites Institutional Review Board (IRB). More information about the ADNI investigators is given in Acknowledgement.

In this study, 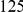 normal control subjects and 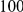 P-MCI subjects are taken from the ADNI dataset. Each subject is rescanned and re-evaluated every six months for up to 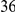 months. The P-MCI subjects are those who developed probable AD after the baseline scanning. The diagnosis of AD is made according to the NINCDS/ADRDA criteria [24] for probable AD. The demographic and clinical information of all the selected subjects are summarized in Table 1.

**Table 1:** Demographic information of the subjects involved in the study.

	Normal Control	P-MCI
No. of Subjects	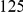	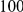
No. 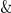 Percentage of males	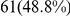	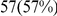
Baseline age, mean(STD)	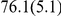	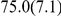
Baseline MMSE, mean(STD)	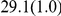	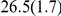

### Image Preprocessing

The T1-weighted MR brain images are skull-stripped and cerebellum-removed after a correction of intensity inhomogeneity using N3 algorithm [25]. Then each MR brain image is further segmented into three tissue types, namely GM, WM, and CSF. To compare structural patterns across subjects, the tissue-segmented brain images are spatially normalized into a template space (called the stereotaxic space) by a mass-preserving registration framework proposed in [26]. During image warping, the tissue density within a region is increased if the region is compressed, and vice versa. These tissue density maps reflect the spatial distribution of tissues in a brain by taking into consideration the local tissue volume prior to warping. After spatial normalization, we can then measure the volumes of GM, WM, and CSF in each predefined ROI. More details about the ROI hierarchy are given in Section “Hierarchical ROI Construction”.

### Method Overview

**Figure 1:**
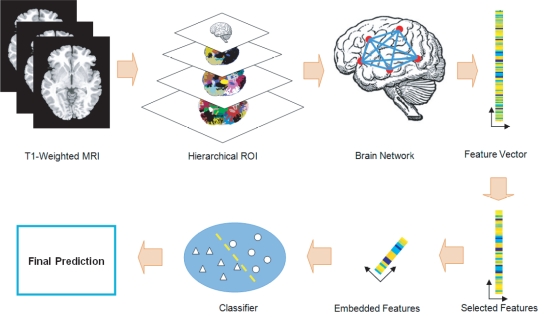
Overview of our proposed method.

The overview of the proposed method is illustrated in Fig. 1. Each brain image is parcellated in multi-resolution according to hierarchically predefined ROIs. The local volumes of GM, WM, and CSF are then measured within these ROIs and used to construct an anatomical brain network. Each node of the network represents an ROI, and each edge represents the correlation of local tissue volumes between two ROIs. The edge values (the correlations) are concatenated to form the feature vectors for use in classification. This gives rise to a large amount of features. For a robust classification, both feature selection and feature embedding algorithms are used to remove many noisy, irrelevant, and redundant features. Only essentially discriminative features are kept to train our classifier that can be well generalized to predict previously unseen subjects. In the following, the description of the proposed method is divided into three parts: hierarchical ROI construction (Section “Hierarchical ROI Construction”), feature extraction (Section “Feature Extraction”), and classification (Section “Classification”).

### Hierarchical ROI Construction

**Figure 2:**
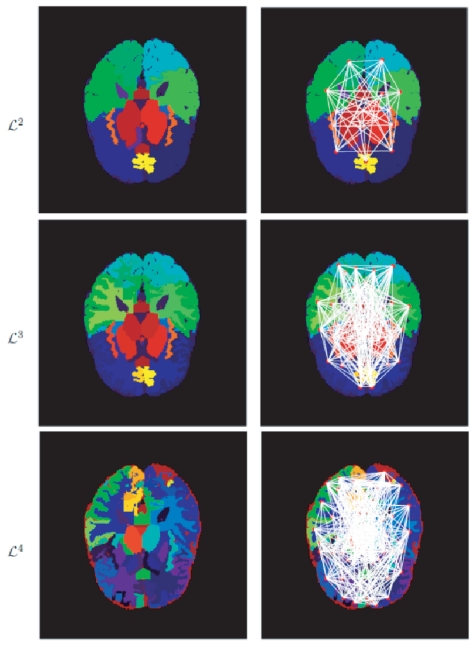
Illustration of hierarchical ROIs. Left: Hierarchical ROIs in three different layers; Right: Network connections between ROIs within different layers.

In this paper, a four-layer ROI hierarchy is proposed to improve the classification performance of volumetric measurements. Each layer corresponds to a brain atlas with different resolution. To make the explanation of our method clear, the bottommost layer that contains the finest ROIs is denoted as 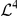, while the other three layers are denoted as 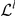, where 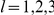. A smaller 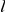 denotes a coarser ROI which is in a layer closer to the top of the hierarchy. In our approach, the bottommost layer 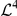 contains 100 ROIs obtained according to [27]. These ROIs include fine cortical and subcortical structures, ventricle system, cerebellum, brainstem, etc. Note that in our case, the cerebellum and the brainstem are removed and the respective ROIs are not actually used. The number of ROIs reduces to 44 and 20, respectively, in the layers 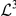 and 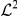 by agglomerative merging of the 100 ROIs in the layer 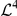. In the layer 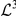, the cortical structures are grouped into frontal, parietal, occipital, temporal, limbic, and insula lobe in both left and right brain hemispheres. Each cortical ROI has three sub-ROIs, namely the superolateral, medial and white matter ROIs. The subcortical structures are merged into three groups in each hemishphere of the brain, namely, the basal ganglia, hippocampus and amygdala (including fornix), and diencephalon. Other ROIs include the ventricle and the corpus callosum. In the layer 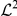, the sub-groups of the superolateral, medial or white matter parts within each cortical ROI are merged together. All the subcortical ROIs are grouped into one ROI. Other ROIs remain the same in the layer 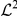 as in the layer 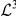 . The topmost layer 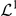 contains only one ROI, the whole brain. This layer 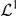 is included because when correlated with the ROIs in 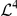, it gives us a measurement comparable to the original volumetric measurements, thus allowing us to also include the original volumetric features for classification. The ROIs for different layers are shown in Fig. 2 (a). The number of ROIs in each layer of the hierarchy is illustrated in Table 2.

**Table 2:** Number of ROIs in the hierarchy.

Layer	Number of ROIs
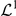	1
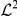	20
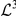	44
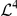	100

### Feature Extraction

**Figure 3:**
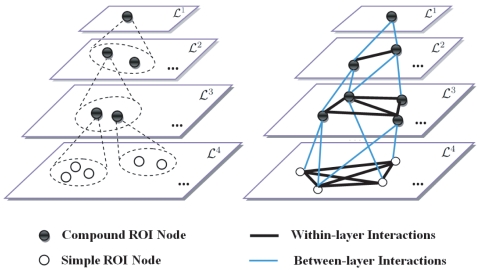
Explanation of the network model. Left: Two types of nodes are included in the hierarchical network: the simple node in 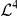, and the compound node in 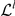
(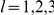). Each compound node is obtained by grouping several simple nodes agglomeratively. Right: Two types of edges are included in the hierarchical network, each modeling the within-layer and between-layer interactions, respectively.

With the ROI hierarchy defined above, an anatomical brain network can be constructed for each subject, from which informative features are extracted for classification. For each brain network, its nodes correspond to the brain ROIs, and its undirected edges correspond to the interactions between two ROIs. There are two types of nodes in our model (Fig. 2-left): the simple ROI in the bottommost layer 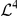, and the compound ROI in the other layers. Similarly, we have two types of edges, each modelling within-layer and between-layer ROI interactions, respectively (Fig. 3-right).

The brain network may be quite complicated. For instance, Fig. 2 (b) partially shows the network connections between ROIs in the layers of 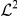, 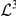 and 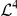, respectively. To determine informative features from the network, the computation of ROI interactions is initially conducted on the bottommost layer 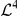, and then propagated to other layers effectively via a membership matrix that indicates the relationship of ROIs from different layers. The process is detailed as follows.

Firstly, let us consider the bottommost layer 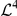, which consists of 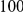 ROIs. Let 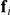 denote the 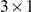 vector of the 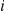-th ROI in 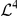, consisting of the volumetric ratios of GM, WM, and CSF in that ROI. We can obtain an 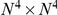 matrix 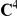, where 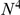 is the number of ROIs in 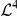. The (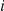, 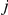)-th component in 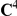 corresponds to the weight of the edge between the 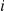-th node and the 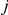-th node in 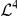. We define 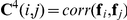, the Pearson correlation between feature vectors 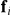 and 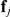.

**Figure 4:**
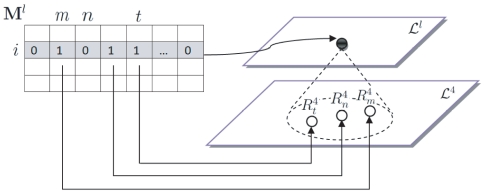
Explanation of the membership matrix. The 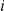-th row in the membership matrix 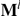 represents the composition of the node 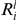 in 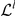. In our example, since 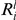 is composed of the simple nodes 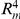, 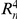 and 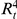 in 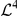, the elements of 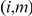, 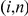 and 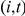 in 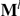 are set to 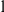, while the others in the 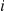-th row are set to 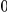.

For any other layer 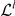, let 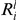 represent the 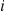-th ROI in the layer 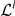. The number of ROIs in the layer 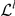 is denoted as 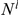. A membership matrix 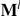 (Fig. 4) is used to define the composition of the compound ROI 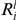 in 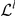. The matrix 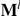 has 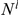 rows and 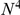 columns. Each row corresponds to a single compound ROI in 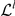. Each column corresponds to a single simple ROI in 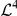. The (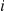, 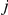)-th component of 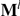 takes the value of either 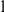 or 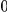, indicating whether the 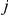-th ROI in 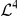 is included in the 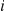-th ROI in 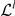. Take Fig. 4 for example. If the ROI 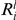 is composed of the simple nodes 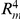, 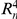 and 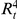 in 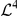, the elements of 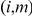, 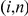 and 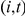 in 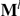 are set to 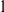, while the others in the 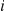-th row are set to 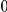. In particular, for the whole brain in 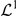, the membership matrix 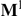 is a row vector with all 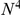 elements set to 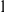. The following shows that the within-layer and between-layer ROI interactions can be calculated by simply performing some linear operations on the matrix 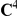 based on the membership matrix 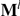.

#### Within-layer ROI interaction

Given the ROI interactions in the bottommost layer 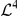, the ROI interactions within each of the higher layers are computed as follows. Let 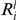 and 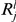 represent the 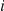-th and 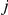-th ROIs in a certain layer 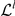. Again, a matrix 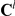 is defined similar to 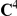, but its (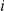, 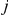)-th component now indicates the correlation between the compound ROIs 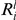 and 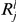. Suppose 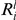 and 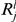 contain 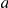 and 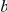 simple ROIs respectively. The correlation between 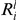 and 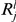 is computed as the mean value of all the correlations between a simple ROI node from 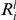 and a simple ROI node from 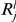, that is,
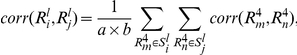
where 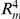 and 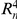 represent the simple ROIs in 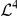, and 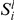 and 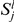 are two sets containing the simple nodes that comprise 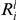 and 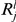, respectively.

Represented in the form of matrix, the correlation matrix 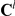 can be computed as follows:
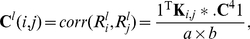
(1)where 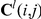 denotes the 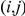-th element in the matrix 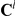, the vector 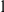 is the 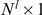 vector with all elements equal to 1, the symbol 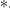 represents component-wise product of two matrices, and the 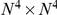 matrix 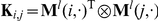 is the Kronecker product of the 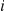-th and the 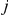-th rows in the membership matrix 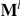.

#### Between-layer ROI interaction

The correlation matrix that reflects between-layer interactions can be defined similarly to that of within-layer interactions. First, let us consider the correlation matrix for two different layers 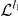 and 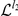 (where 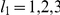; 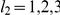; and 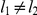). It is defined as:
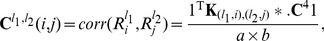
(2)where 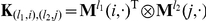 is the Kronecker product of the 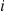-th row in 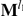 and the 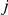-th row in 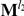.

Now, let us consider the correlation matrix for two layers 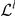 and 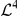. It can be simply computed as:
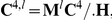
where 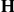 is an 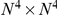 matrix, whose elements in the 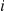-th row are all equal to 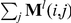, and the symbol 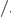 denotes the component-wise division of two matrices.

#### Feature vector construction

Note that the hierarchical anatomical brain network may not have the property of small-worldness as shown in DTI and fMRI networks [28], [29], because the connections in our case are not based on functions or real neuron-connections. Some prior knowledge could be used to prune the edges if it is believed that two ROIs are independent of each other conditioned on the disease. However, in our approach we keep all the connections so that new relationships between structural changes and the disease are not left unexplored. But on the other side, since our network is fully connected, some commonly used network features, such as local clustering coefficients, do not work efficiently as they do for sparse networks in DTI and fMRI. The local clustering coefficient for a node 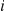 is computed by averaging its connections to all the other nodes in the network, which might eliminate the necessary discrimination. Therefore, we directly use the weights of edges as features, that is, we concatenate the elements in the upper triangle matrices of correlation matrices computed above. Moreover, before computing the correlation of the volumetric features 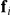 and 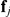, we employ a normalization step by subtracting 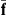 from 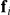, where 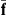 is the mean volume (in GM, WM, and CSF) of different ROIs belonging to the same subject. By centerizing features in this way, we can obtain a better classification accuracy.

### Classification

**Figure 5:**
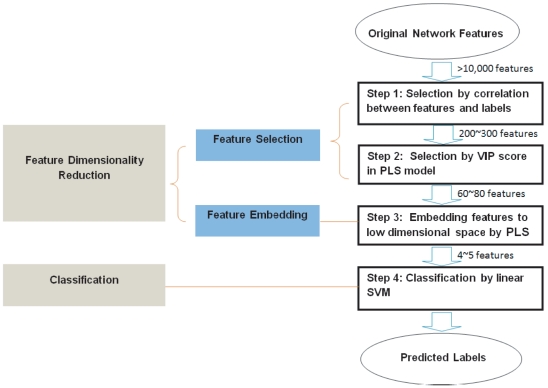
Overview of the proposed classification scheme.

Since a hierarchical fully-connected brain network is used in our study, the dimensionality of the network features is very high: originally more than 10,000 features for each subject. To address this issue, in this paper, we propose a classification scheme to efficiently learn discriminative information from this large amount of network features. The scheme involves feature dimensionality reduction and classification. The overview of the whole process is given in Fig. 5. As shown, we use both a two-step feature selection (Step 1 and Step 2 in Fig. 5) and a feature embedding (Step 3 in Fig. 5) algorithms to efficiently reduce the dimensionality of features. This gives rise to a small number of discriminative features that can be well separated by a linear classifier. In particular, the features of the training subjects are first selected according to their relevance with respect to the clinic labels. This step reduces the original more than 10,000 features to about 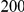 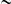 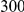 features. Then in the second step, about 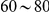 features are further selected based on their predictive power in a Partial Least Square (PLS) model [30]. After the two-step feature selection, another PLS model is trained to embed the selected 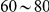 features into a low dimensional space that maintains their discriminative power. After feature selection and feature embedding, each subject is represented by only 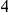 to 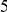 features. These features are fed into a linear SVM classifier for differentiating MCI patients and normal controls (Step 4 in Fig. 5).

In the rest of this section, our proposed classification scheme is explained in detail. Firstly, in Section “Problem on identifying discriminative features”, we justify the necessity of incorporating both feature selection and feature embedding into the dimensionality reduction module in Fig. 5. Then a brief introduction about the Partial Least Square analysis is given in Section “Partial Least Square analysis”, which is the key technique used in our classification scheme. PLS integrates the dimensionality reduction process (Step 1 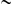 3 in Fig. 5) and the classification process (Step 4 in Fig. 5) by considering classification labels when seeking a low dimensional embedding space. It also integrates feature selection (Step 2 in Fig. 5) and feature embedding (Step 3 in Fig. 5) into the same framework to optimize the selection performance. Finally, we summarize how PLS is used to facilitate the classification in our case step by step in Section “Summary of the proposed classification scheme”.

#### Problem on identifying discriminative features

When the number of predefined ROIs is large, the traditional volumetric-based approaches encounter the high feature dimensionality problem. Therefore some preprocessing steps are conducted to reduce the feature dimensionality before classification. There are usually two ways: i) select a subset of the most discriminative features from the original feature set, known as feature selection, or ii) combine the original features linearly or non-linearly to get a lower dimensional feature space, known as feature embedding. Both methods have been reported in the literature. In [4], [5], a small subset of features are selected by SVM-Recursive Feature Elimination (SVM-RFE) proposed in [31] and then fed into a nonlinear SVM with a Gaussian kernel. In [32], the volumetric feature vector concatenating the GM, WM and CSF in ROIs are nonlinearly embedded into a lower dimensional feature space by Laplacian Eigenmap, and then a clustering method is used to predict the AD from the normal control.

Compared with volumetric features, the dimensionality of our proposed network features is even much higher. To address this problem, we propose to use both feature selection and feature embedding to efficiently reduce the feature dimensionality. The reason is two-fold. Firstly, feature selection alone may still give rise to many informative features for the classification. For example, suppose that only 10 ROIs really contribute to the discrimination. The dimension of volumetric features may be maximally reduced to 10 if the feature selection method is effective. However, the number of the corresponding network features that model the pairwise interactions of the 10 ROIs might be up to 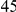. This possible number is only computed for the layer 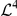. If considering about the interactions of ROIs between different hierarchical layers, this number will be further increased. Secondly, feature embedding based on the original high dimensional features may not be able to accurately estimate the underlying data structure due to the existence of too many noisy features. Also, a large amount of features will greatly burden the computation of embedding.

In short, either feature selection or feature embedding *alone* may not be sufficient to identify the discriminative network features with respect to classification. Therefore, a dimensionality reduction process is proposed, which couples feature selection and feature embedding via Partial Least Square (PLS) analysis [30]. As a supervised learning method, PLS considers about the information in the classification labels and thus achieves a better discrimination than many of the commonly used unsupervised methods, for example, Principal Components Analysis (PCA) and the Laplacian Eigenmap. As the key technique used in our classification scheme, a brief introduction about PLS is given to make our paper self-contained.

#### Partial Least Square analysis

PLS models the relations between the predictive variables (the features 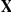) and the target variables (the labels 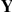) by means of latent variables. It is often compared to PCA that only models the eigenstructure of 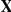 without considering the relationship between 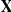 and 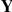. PLS maximizes the covariance of the projections of 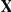 and 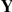 to latent structures, as well as the individual variance of 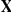 and 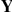. This method has advantages on data set where the size of the samples is much smaller than the size of the features.

In particular, let the 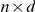 matrix 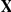 represent the 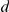-dimensional feature vectors for the 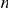 subjects, and 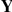 represent the corresponding 1-dimensional label vector. PLS decomposes the zero-mean matrix 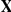 and the zero-mean vector 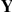 into
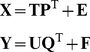
(3)where 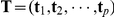 and 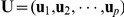 are 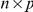 matrices containing 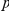 extracted latent vectors, the 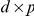 matrix 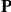 and the 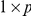 vector 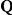 represent the loadings, and the 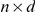 matrix 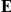 and the 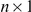 vector 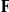 are the residuals. The latent matrices 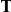 and 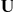 have the following properties: each column of them, called a latent vector, is a linear combination of the original variables 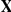 and 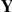, respectively; and the covariance of two latent vectors 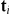 and 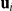 is maximized. PLS can be solved by an iterative deflation scheme. In each iteration, the following optimization problem is solved:
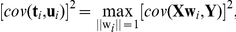
where 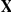 and 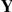 are deflated by subtracting their rank-one approximations based on 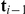 and 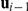. Once the optimal weight vector 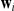 is obtained, the corresponding latent vector 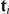 can be computed by 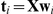. For more details, please see [30].

#### Summary of the proposed classification scheme

Taking advantages of PLS analysis, our proposed method achieves good classification and generalization in four steps, as shown in Fig. 5.

*In Step 1*, the discriminative power of a feature is measured by its relevance to classification. The relevance is computed by Pearson correlation between each original feature and the classification label. The larger the absolute value of the correlation, the more discriminative the feature. Features with correlation values lower than a threshold are filtered out.

*In Step 2*, a subset of features are further selected from the result of Step 1 in order to optimize the performance of PLS embedding in Step 3. In particular, a PLS model is trained using the selected features from Step 1. Then a method called Variable Importance on Projection (VIP) [33] is used to rank these features according to their discriminative power in the learned PLS model. The discriminative power is measured by a VIP score. The higher the score, the more discriminative the feature. A VIP score for the 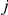-th feature is
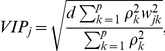
where 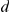 is the number of features, 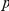 is the number of the latent vectors as defined above, 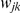 is the 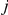-th element in the vector 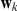, and 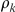 is the regression weight for the 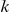-th latent variable, that is, 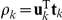. About 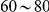 features with the top VIP scores are selected for feature embedding in the next step.

*In Step 3*, using the features selected in Step 2, a new PLS model is trained to find an embedding space which best preserves the discrimination of features. The embedding is performed by projecting the feature vectors in the matrix 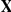 onto the new weight vectors 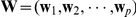 learned by PLS analysis. In other words, the representation of each subject changes from a row in the feature matrix 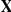 to a row in the latent matrix 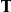. The feature dimensionality is therefore reduced from 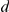 to 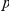 (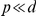).

*In Step 4*, after PLS embedding, a small number of features in the new space are able to capture the majority of the class discrimination. This greatly reduces the complexity of relationships between data. Therefore, these features are used to train a linear SVM for predicting MCI patients and normal controls. In our case, a linear SVM can achieve better or at least comparable classification accuracies as a non-linear SVM.

The advantages of PLS for our network features over some commonly used unsupervised and supervised nonlinear methods, such as Laplacian eigenmap embedding and Kernel Fisher Discriminant Analysis (KFDA), have been evidently shown in our experiment in Section “Comparison of Classifiers”.

## Results and Discussion

In our study, we conduct two kinds of comparisons, that is, to compare the discrimination power of the network and the volumetric features, and to compare the performance of different classifiers for the network features. The discussion of the classification results are given at the end of this section.

Please note that, as MCI patients are highly heterogeneous, the comparison of the absolute classification accuracy with the existing works in the literature is meaningless. Therefore in our study, we evaluate the improvement of our proposed approach over the conventional volumetric features by comparisons on the same data set with the same experiment configuration. Furthermore, to investigate the generalization of the proposed method, we conduct experiments repetitively on different random partitions of training and test data sets with different partition ratios. The average classification accuracy estimated in this way tends to be more conservative than the traditional Leave-One-Out approach. More discussions are given below.

### Comparison of Features

Firstly, we compare the efficacy of different features with respect to classification. The data set is randomly partitioned into 20 training and test groups with 75 samples for training and 75 samples for test. For a fair comparison, our proposed classification process is applied similarly to both the volumetric and the network features.

As aforementioned, our network features differ from the conventional volumetric features in two aspects: i) the network features model the regional interactions; ii) the network features are obtained from a four-layer hierarchy of brain atlases. The contributions of these two aspects are investigated separately. To test the advantages of using regional interactions over local volumes, we compare the network and the volumetric features on the same hierarchical structure (either single-layer or four-layer). To test the advantages of using the hierarchical network structure, we compare network features obtained from different layers (the bottommost layer and all four layers) in the hierarchy. Moreover, we compare the networks with and without the cross-layer connections to further explore the function of the hierarchial structure. In summary, five methods are tested in the experiment:

- Method I is the proposed method in this paper, using the four-layer hierarchical network features.
- Method II only uses the network features from the bottommost layer 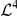. It tests the classification performance of network features on a single layer.
- Method III uses the network features from all the four layers, but removing the edges across different layers. It tests how the cross-layer connections in the hierarchy contribute to the classification.
- Method IV uses the volumetric features (the concatenation of GM, WM and CSF ratios in the ROIs) from the bottommost layer 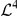. It corresponds to the conventional volume-based method.
- Method V uses volumetric measures from all four layers. It tests if the volumetric features obtained from the hierarchy can achieve similar classification performance as the hierarchical network features.

**Figure 6:**
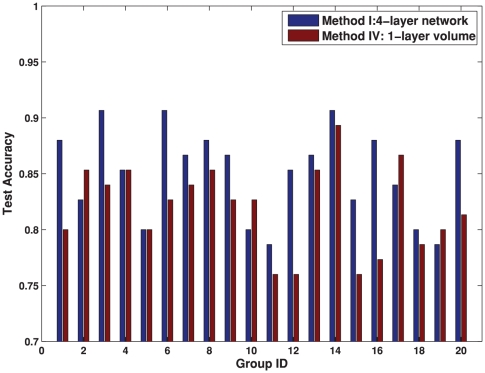
Classification comparison using different features. The classification performance is compared between our proposed method (four-layer network features as in Method I) and the conventional volumetric method (Method IV) on 20 training/test groups. Each group contains 150 training samples and 75 test samples randomly partitioned from our data set.

**Figure 7:**
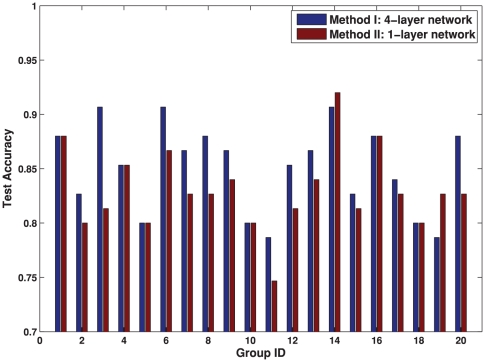
Classification comparison using different hierarchical structure. The classification performance is compared between the four-layer network features in Method I and the single layer network features in Method II on 20 training/test groups. Each group contains 150 training samples and 75 test samples randomly partitioned from our data set.

The results are summarized in Table 3. The classification accuracy in Table 3 is averaged across the 20 randomly partitioned training and test groups. A paired 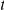-test is conducted between Method I and the other four methods respectively, to demonstrate the advantage of our proposed method. The 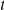-value and the corresponding 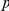-value of the paired 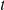-test are also reported. It can be seen from Table 3 that Method I is always statistically better (the significance level 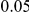) than any of the other four methods. In addition to comparing the average accuracies in Table 3, the classification accuracies are also compared on each of the 20 training-test groups between the four-layer network features (Method I) and the conventional volume features (Method IV) in Fig. 6, and between the four-layer network features (Method I) and the single-layer network features (Method II) in Fig. 7.

**Table 3:** Comparison of discrimination efficacy of different features.

	Mean Test Accuracy ( 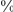 )	Paired 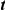 -test	
		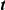 -value	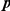 -value
Method I	85.07 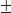 3.92	-	-
Method II	83.0 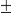 3.65	3.1349	0.00272
Method III	83.13 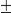 3.43	3.0009	0.00367
Method IV	81.93 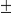 3.76	3.3558	0.00166
Method V	81.47 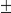 3.95	4.4163	0.00015

Combining the results from Table 3, Fig. 6 and Fig. 7, we observe the following:

- Our proposed hierarchical network features in Method I outperform the conventional volumetric features in Method IV. The advantage may come from using both regional interactions and the hierarchical structure.
- To demonstrate the benefit purely from using the regional interactions, the same atlases in the hierarchy are applied to volumetric features as in Method V. It can be seen from Table 3 that the hierarchical structure does not improve the discrimination of the single-layer volumetric features in Method IV. Moreover, the benefit of using regional interactions can also be shown by the better result of the single-layer network features in Method II than the single-layer volumetric features in Method IV.
- To demonstrate the benefit purely from the hierarchy, we compare the classification performance of the single-layer network features in Method II and the four-layer network features in Method I. The advantage of the four-layer structure is statistically significant over the single-layer. Moreover, the result that Method I statistically outperforms Method III indicates the necessity of using the cross-layer edges in the network.

**Figure 8:**
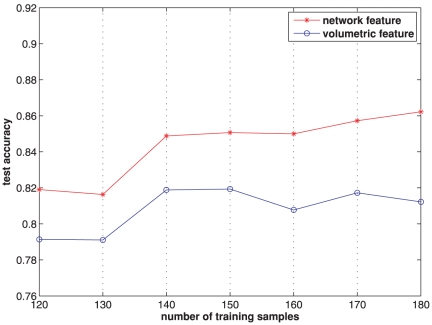
Classification comparison using network features and volumetric features with different numbers of training samples.

It is noticed that different ratios of training and test partitions may lead to a variation in the classification accuracy. To reflect the influence of this factor, we test seven different numbers of training samples, occupying 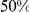 to 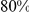 of the total data size. For each number of training samples, 20 training and test groups are randomly generated and the average classification accuracy is summarized in Fig. 8. When 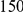 training samples are used, the test accuracy in Fig. 8 corresponds to the classification accuracy of 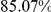 obtained by Method I in Table 3. In general, the classification accuracy goes up slightly when the number of the training samples increases. This is not surprising because the larger the number of training samples, the more the learned information. It can be seen that the network features show a consistent improvement in classification accuracy of approximately 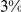 in all cases, compared to those by using the conventional volumetric features. Averaged across different numbers of training samples, the classification accuracy becomes 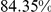 for the network features, and 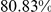 for the volumetric features, which represents an overall classification performance of these two different features. A paired 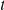-test is performed on the seven different ratios of training-test partitions using both features. The obtained 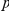-value of 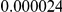 indicates that the improvement of the network features over the volumetric features is statistically significant.

It is worth noting that the influence of different ratios of training-test partitions on the classification result is often ignored in many existing works. One possible reason is that a Leave-One-Out validation is used when the size of the data is small. This often leads to the use of more than 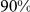 data for training, which tends to produce a more optimistic result compared with using other lower ratios of training data.

### Comparison of Classifiers

The classification performance of our proposed classification scheme is compared with other six possible schemes shown in Table 4. To simplify the description, our proposed scheme is denoted as P1, while the other six schemes in comparison are denoted as P2
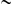
P7. To keep consistent with P1, each of the six schemes P2
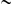
P7 is also divided into four steps: rough feature selection, refined feature selection, feature embedding and classification, corresponding to Step 1
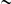
Step 4 in P1. Please note that the first step, rough feature selection, is the same for all schemes P1
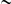
P7. In this step, the discriminative features are selected by their correlations with respect to the classification labels. From the second step onwards, different schemes utilize different configurations of strategies, as shown in the second column of Table 4.

**Table 4:** Configurations of classification Schemes.

Schemes	Configurations	classification accuracy overall ( 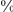 )
P1	VIP selection + PLS embedding + linear SVM	84.35
P2	VIP selection + PLS embedding + nonlinear SVM	84.03
P3	no selection + PLS embedding + linear SVM	84.11
P4	no selection + PLS embedding + nonlinear SVM	84.10
P5	SVM-RFE selection + no embedding + nonlinear SVM	80.07
P6	no selection + Laplacian Eigenmap embedding + nonlinear SVM	79.16
P7	no selection + KFDA embedding + linear SVM	81.08

To clarify the settings of our experiment, the Laplacian embedding used in P7 is described as follows. The embedding is applied on a connection graph that shows the neighboring relationship of the subjects. Based on the connection graph, the distance between two subjects is computed as the shortest distance between the corresponding two nodes in the graph. This distance is used to construct the adjacent matrix and Laplacian matrix used in the Laplacian embedding. The Laplacian embedding in our experiment is different from the one in [32] where the distance between two subject is computed based on the deformation estimated by the registration algorithm.

**Figure 9:**
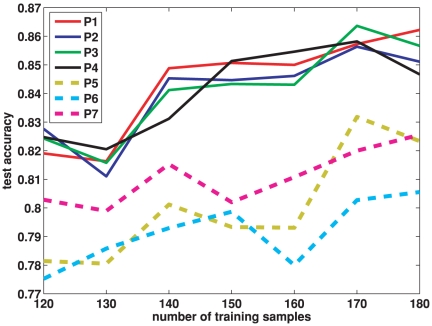
Comparison of seven classification schemes on network features. The classification accuracy is plotted over different number of training samples. For a given number of training samples, the classification accuracy is averaged over 20 training/test groups randomly partitioned from our data set using this number of training samples. The scheme configurations are shown in Table 4.

The classification results are summarized in Fig. 9 and Table 4. Please note that the classification accuracy at each number of training samples in Fig. 9 is an average over 20 random training and test partitions as mentioned in Section “Comparison of Features”. Also, the overall classification accuracy in Table 4 is an average of accuracies at different numbers of training samples in Fig. 9. The best overall classification accuracy of 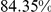 is obtained by our proposed scheme P1: VIP selection + PLS embedding + a linear SVM. This is slightly better than P2, where a nonlinear SVM is used. It can be seen that the classification schemes with PLS embedding (P1
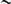
P4) achieve an overall accuracy above 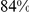, better than those without PLS embedding (P5
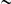
P7). The supervised embedding methods, i.e., PLS (P1
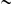
P4) and KFDA (P7), perform better than the unsupervised Laplacian Eigenmap embedding (P6). Moreover, PLS embedding (P1
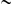
P4) preserves more discrimination than the nonlinear supervised embedding of KFDA (P7).

Although the proposed scheme P1 achieves the best classification performance, the difference between P1
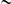
P4 is not significant. This may indicate that the discriminative dimensionality reduction by PLS embedding plays a more important role than the classifier type in improving classification performance. After PLS embedding, the data complexity is greatly reduced and the intrinsic relationship underlying the data becomes more evident, therefore allowing even simple classifiers to achieve performance comparable to more sophisticated classifiers. Although the difference between P1
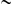
P4 is not significant, P1 is still preferred over P2 and P4 because the linear SVM employed in P1 is much faster than the nonlinear SVM employed in P2 and P4. P1 is also preferred over P3, because the VIP selection employed in P1, while yielding improvement over P3, does not increase the computational cost substantially.

### Spatial Patterns

Note that each network feature characterizes the relationship between two ROIs, instead of an individual ROI as in the conventional approaches. Therefore, for the first time, we study the *relative* progression speed of the disease in different ROIs of the same subject, which eliminates the impact of personal variations. On the contrary, the conventional methods study the *absolute* progression speeds of ROIs among different subjects. Normalizing subjects by the whole brain volume in conventional methods may not completely remove the personal variations.

To be an essentially discriminative network feature, the two associated ROIs may satisfy one of the two following conditions:

- One ROI shows significant difference between the MCI group and the normal control group, while the other ROI is relatively constant with respect to the disease. Therefore the correlation between these two ROIs varies over the two groups in comparison.
- Both ROIs change with the disease, but their change speeds are different over two different groups.

The selected features are different for the twenty randomly partitioned training and test groups used in Section “Comparison of Features”. Table 5 shows the most discriminative features selected by more than half of the training and test groups. It can be clearly seen that hippocampus remains the most discriminative ROI in differentiating the normal controls and MCI patients. Table 5 is separated into two parts. On the upper portion of the table, the two ROIs of a network feature may be both associated with the MCI diagnosis, such as hippocampus, entorhinal cortex, uncus, fornix, globus palladus, cingulate etc, as reported in the literature [4], [5], [7], [10], [13], [15], [19], [20], [34], [35]. A typical example is the correlation between hippocampus and ventricle. It is known that the enlargement of ventricle is a biomarker for the diagnosis of the AD [36]. However, different from the hippocampus volume loss that often occurs at the very early stage of the dementia, the ventricle enlargement often appears in the middle and late stages. Therefore, the progression pattern of disease in these two regions is different. Their correlation is thus selected as the discriminative feature. On the lower portion of the table, the first ROI is associated with the disease, while the second ROI is not. For example, it has been reported that the anterior and posterior limbs of internal capsule and the occipital lobe white matter are not significantly different between MCI patients and normal controls in a DTI study [37].

**Table 5:** Selected discriminative features.

hippocampus – amygdala
hippocampus - lingual gyrus
hippocampus – uncus
hippocampus - prefrontal/superolateral frontal lobe
hippocampus - globus palladus
hippocampus - entorhinal cortex
hippocampus - cingulate region
hippocampus – ventricle
hippocampus and amygdala and fornix – ventricle
uncus – fornix
hippocampus - posterior limb of internal capsule
globus palladus - anterior limb of internal capsule
hippocampus - occipital lobe WM

### Metrics

In our network design, each edge represents the correlation or the “similarity” between a pair of ROI nodes. Pearson correlation is just one of the possible similarity measurements. By viewing Pearson correlation as an inverse distance, it is straightforward to include other commonly used distance metrics, e.g., the Euclidean distance, the 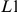-norm distance, and the kernel based distance, for measuring the feature similarity between ROI pairs. By virtue of separating the computation of the hierarchy and the regional interactions, our proposed method can be easily generalized to other metrics with merely a slight revision of (1) and (2) as follows. The within-layer interaction is computed as
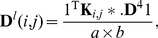
(4)and the between-layer interaction is computed as
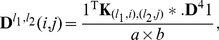
(5)where 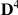 is a general metric that measures the relationship between two ROIs in the bottommost layer 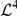. The definitions of other symbols remain the same. If Pearson correlation is used, these two equations become identical to (1) and (2). It can be seen that, for a different metric, the hierarchy can be left intact and only the regional interactions in the bottommost layer need to be recomputed.

**Figure 10:**
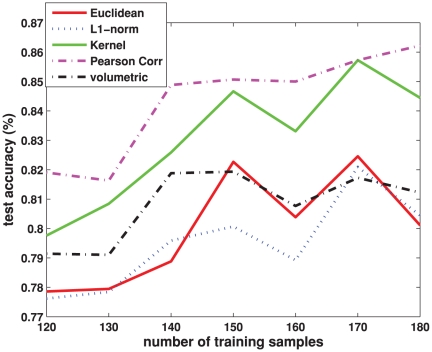
Comparison of different metrics used for modeling the regional interactions. The classification accuracy is plotted over different number of training samples. For a given number of training samples, the classification accuracy is averaged over 20 training/test groups randomly partitioned from our data set using this number of training samples.

Using (4) and (5), we test the performance of the three alternative metrics: the Euclidean distance 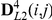, the 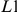-norm distance 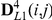, and the kernel based distance 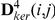. They are defined as follows:
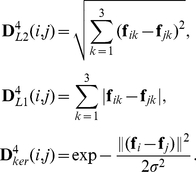
(6)The Euclidean distance and the 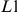-norm distance measure the linear relationship between a pair of ROI nodes. No parameter needs to be set. The kernel based distance provides a non-linear measurement of ROI feature similarity. The parameter 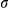 is set, by cross-validation, to be 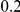 times the average Euclidean distance between ROI pairs. Based on the 20 random training and test partitions as in Section “Comparison of Features”, the average classification accuracies are reported in Table 6. For comparison, the accuracies of our network approach using Pearson correlation, and the conventional volumetric approach are also repeated in the table. In addition, the test accuracies over different numbers of training samples for different metrics are plotted in Fig. 10. It can be seen that, Pearson correlation yields the best performance, followed by the kernel based distance. These two distances give significant improvement over the conventional volumetric approach, whereas the Euclidean and the 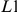-norm distances do not. The importance of the choice of the metric is quite visible: only when a proper metric is selected, the network construction may bring useful information compared with the conventional volumetric approach.

**Table 6:** Comparison of different metrics for modeling the regional interactions.

	Mean Test Accuracy ( 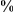 )
Euclidean	82.27
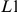	80.07
Kernel	84.47
Pearson Correlation	85.07
Volumetric	81.93

### Conclusion

In this paper, we have presented how hierarchical anatomical brain networks based on T1-weighted MRI can be used to model brain regional correlation. Features extracted from these networks are employed to improve the prediction of MCI from the conventional volumetric measures. The experiments show that, without requiring new sources of information, the improvement brought forth by our proposed approach is statistically significant compared with conventional volumetric measurements. Both the network features and the hierarchical structure contribute to the improvement. Moreover, the selected network features provide us a new perspective of inspecting the discriminative regions of the dementia by revealing the relationship of two ROIs, which is different from the conventional approaches. The flexibility to generalize our proposed method has been demonstrated by different distance metrics tested in our experiment.
